# Comparison of Vessel Sealing Systems with Conventional

**DOI:** 10.5812/ircmj.10180

**Published:** 2013-06-05

**Authors:** Kemal Peker, Abdullah İnal, Huriye Güllü, Düriye Gül, Murat Şahin, Ayca Dumanli Ozcan, Kemal Kılıç

**Affiliations:** 1 Erzincan University Department of General Surgery, Erzincan, Turkey; 2 Erzincan University Department of Anesthesiology & Reanimation, Erzincan, Turkey; 3 Palandoken State Hospital Department of Anesthesiology & Reanimation, Erzurum, Turkey; 4 Kafkas University Department of General Surgery, Kars, Turkey

**Keywords:** Haemorrhoidectomy, LigaSure, Harmonic Scalpel

## Abstract

**Background:**

Haemorrhoids are cushions of submucosal vascular tissue located in the anal canal starting just distal to the dentate line. Haemorrhoidal disease is a common anorectal disorder which has symptoms of bleeding, prolapse, pain, thrombosis, mucus discharge, and pruritus. Haemorrhoidectomy is one of most frequently performed anorectal operation worldwide.

**Objectives:**

The aim of this study was to compare the effectiveness of the LigaSure tissue sealing device, Harmonic Scalpel and conventional MM open haemorrhoidectomy.

**Materials and Methods:**

Sixty-nine patients with newly diagnosed symptomatic grade three or grade four haemorrhoidal disease, from July 2011 to December 2011 were recruited for the study. Patients were prospectively randomized to LigaSure, Harmonic Scalpel and conventional haemorrhoidectomy. Patients were evaluated on the basis of the mean operative time, postoperative pain, day of discharge, early and late complications.

**Results:**

Each group has twenty-three patients. Ten (14.5 %) were female and fifty-nine (85.5 %) were male. Mean age were 44.5 ± 10.8 for LigaSure group, 39.5±14.4 for Harmonic Scalpel group and 39.8 ± 13.6 for conventional haemorrhoidectomy group. Mean operative time was 12.6 ± 2.9 for LigaSure group, 12.6 ± 2.5 for Harmonic Scalpel group and 22.3 ± 4.5 for conventional haemorrhoidectomy group. Postoperative pain and required analgesic dose were significantly lower for conventional haemorrhoidectomy. Wound healing was also more rapid in conventional haemorrhoidectomy than both LigaSure and Harmonic Scalpel.

**Conclusions:**

Lateral heat dissipation of energy based cautery such as Harmonel Scalpel and LigaSure is considerably high when compared with conventional methods. More thermal damage which is generated on tissue seems to be the reason for increased degree of postoperative pain and delay in wound healing.

## 1. Background

Haemorrhoidal veins are normal components of human anatomy. The disintegration of connective tissue promotes loss of the anatomic relationship with sphincter, resulting in vascular malformations such as varicosis or varicocele (1). A recent study from Austria revealed the prevelance of haemorrhoidal disease as high as 38.93% in general population. In the same study the prevalence of patients who were required surgical treatment was found 8.69% (2). A number of articles discussing for the optimal treatment of haemorrhoidal disease have been published recently; and new devices and procedures have been proposed to overcome the complications due to haemorrhoidectomy (3).

Conventional Milligan-Morgan (MM) open haemorrhoidectomy is still most commonly performed procedure for prolapsing haemorrhoids (4). Also increasing numbers of studies suggest use of the tissue-sealing and ultrasonic devices to provide an alternative to open haemorrhoidectomy for third and fourth degree piles (5, 6). The LigaSureTM(Covidien, Colorado, USA) vessel-sealing system allows complete coagulation of blood vessels up to 7 mm in diameter while confining the thermal spread to within 2 mm of adjacent tissue. This advantage has been extended to the excision of haemorrhoids as it allows fast bloodless dissection with minimal collateral thermal damage (7). The Harmonic ScalpelTM (Ethicon Endo-Surgery, Cincinnati, OH, USA) is an ultrasonically activated instrument which vibrates at a rate of 55,000 MHz. It is known for its ability to coagulate small and medium sized vessels by converting electrical energy to mechanical energy (8, 9). Most of studies suggest that complications are less, degree of pain is lower and amount of analgesic consumption is less for energy based cautery haemorrhoidectomy during post-operative period, and claimed that this method provides a more tolerable postoperative period for patients (10). However, our experience and clinical observations were suggesting otherwise.

## 2. Objectives

The aim of this study was to compare the effectiveness of the LigaSure tissue sealing device, Harmonic Scalpel and conventional MM open haemorrhoidectomy.

## 3. Materials and Methods

This study was approved by the Ethics Committee of the Kafkas University and performed according to the Declaration of Helsinki in 07.07.2011 with code number of 51. The study is conducted in three different cities of Turkey (Kars, Erzurum and Erzincan). All patients were asked to provide written informed consent prior to enrollment, after explanation of the associated risks and benefits and description of the study protocol. All patients were informed that have rights to withdraw or refuse to involve in the study. During a six-month period from July 2011 to December 2011, 69 patients were randomly assigned to LigaSure haemorrhoidectomy (G1), Harmonic Scalpel haemorrhoidectomy (G2) and conventional haemorrhoidectomy (G3), prospectively. All patients had a minimum follow-up period of twelve months (range 12–24) after operation. The overall mean age was 41.3 ± 13.0 (range 19-66) years. Mean age was 44.5 ± 10.8 for group 1, 39.5 ± 14.4 for group 2 and 39.8 ± 13.6 for group 3. Mean operative time was 12.6 ± 2.9 minutes for group1, 12.6 ±2.5 minutes for group2 and 22.3 ± 4.5 minutes for group3. The characteristics of the patient groups are shown in Table 1. 

**Table 1.** Patient Demographics

Variable Value	G1	G2	G3	P
**No of Patients**	23	23	23	
**Mean age, years**	44.56 ± 10.85	39.47 ± 14.41	39.86 ± 13.64	0.345
**Mean duration of disease, mon**	34.95 ± 17.01	33.69 ±17.1	35.65 ± 18.72	0.930
**Mean Operative time, min**	12.67 ± 2.94	12.6 ± 2.5	22.3 ± 4.5	0.000
**Mean no. of soaked gauzes**	0.45 ± 0.91	0.56 ± 0.78	5.04 ± 1.63	0.000
**Sex, No. (%)**				
Male	20 (33.9)	17 (28.8)	22 (37.3)	0.108
Female	3 (30.0)	6 (60.0)	1 (10.0)	
**Haemorrhoid prolapsed, %**	100	100	100	
**Elective surgery, %**	100	100	100	
**Mean no. of piles excised, No. (%)**				
2	6 (26.1)	4 (17.4)	6 (26.1)	0.070
3	11 (47.8)	12 (52.2)	17 (73.9)	
4	6 (26.1)	7 (30.4)		

All patients with newly diagnosed symptomatic third and fourth degree haemorrhoids requiring haemorrhoidectomy were included in the recruitment for the study. Patients complicated with fistula-in-ano, anal fissure, abscess, cancer, inflammatory bowel disease, dermatitis, poor general condition, allergy to any of the standard medication or who did not give consent to the trial were excluded from the study. Four patients have been excluded for having proctologic co-morbidities (anal fissure, dermatitis etc.) and two patients have been excluded for not obtaining a consent. 25 cases were included for control group, 24 cases were included for Harmonic scalpel group and 24 cases were included for Ligasure group. 2 patients from control group, one patient from Harmonic scalpel group and one patient from Ligasure group were excluded for dropping out from follow-up. Patients were evaluated on the basis of the mean operative time, postoperative pain, early and late complications.

### 3.1. Study Design

Indication for operation was determined by a specialist who was blinded to study. Patients enrolled for the study were randomized into three groups by a computer program. The code enclosed in a numbered envelope corresponding to one of the three techniques was shown to the surgeon at the beginning of the operation. All patients were operated by the same surgeon. Wound healing level was evaluated by another specialist who was blinded to the study. A standard medication package was administered to all patients postoperatively.

### 3.2. Surgery

**Figure 1.**
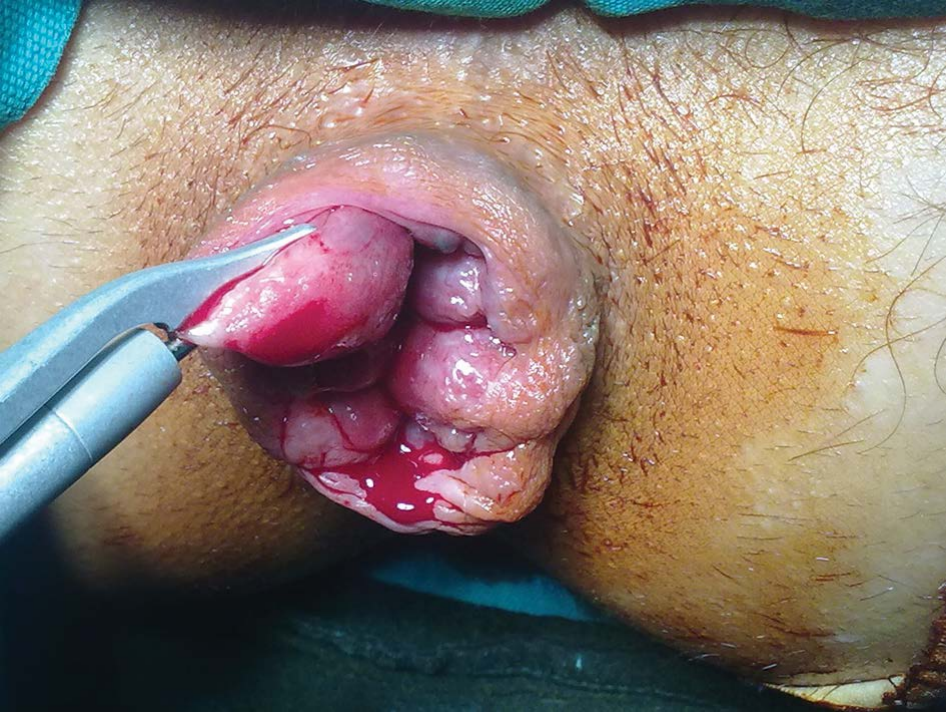
Piles were excised with harmonic scalpel

**Figure 2.**
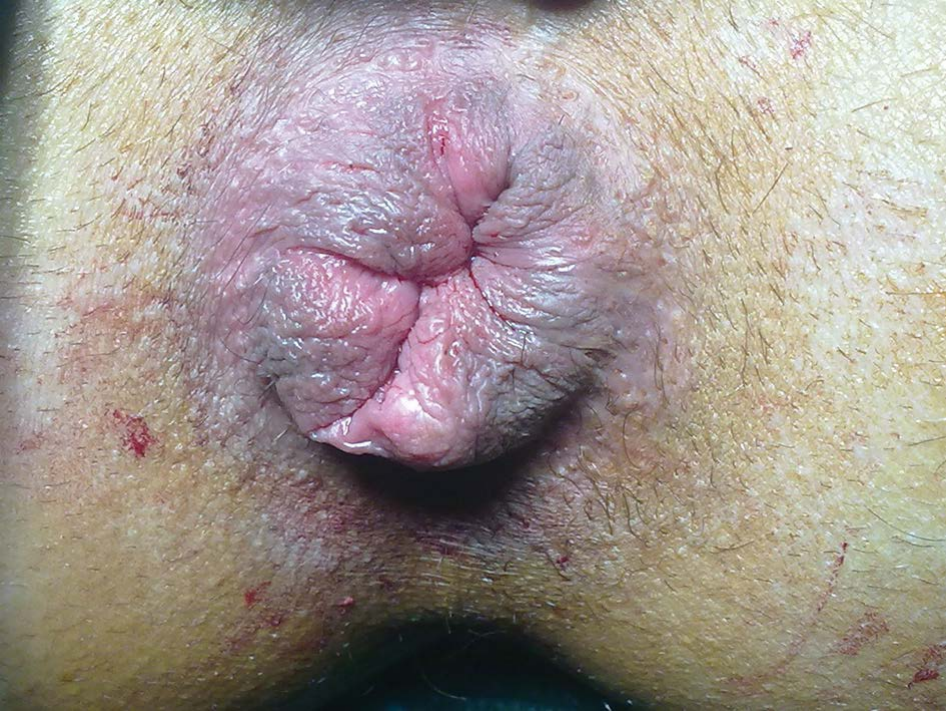
Early view of the anal region after the haemorrhoidectomy with harmonic scalpel

All patients were examined with flexible proctoscopy to exclude other diseases of anorectum before surgery. All patients were applied a 210 ml Sodium Phosphate enema prior to surgery for bowel preparation. No antibiotic prophylaxis had been administered. Spinal anaesthesia was performed in the sitting position at L3-L4 interspace using a 25 Gauge Quincke tip spinal needle. 10 mg 5% bupivacaine was given intrathecally. The patients were placed supine position 2 minutes after spinal anaesthesia. After the adequate dermatomal level of sensory blockage was observed, supine lithotomy position was given to all patients. Patients underwent Milligan-Morgan open haemorrhoidectomy using either LigaSure, Harmonic Scalpel or conventional diathermy (Figure 1). Suture and anal pad was not used in any of patients (Figure 2). 

The operative time was calculated by an operating room nurse, from the beginning of the excision of the first pile until the excision of last pile. Blood loss was recorded as the number of soaked gauze pads.

### 3.3. Postoperative Care

All patients were prescribed sitz baths four times a day for 4 weeks and oral lactulose to aid defecation after the operation. A maximum of 800 mg tramadol was given all patients in first 48 hours by a patient controlled analgesia (PCA) device. If it was inadequate for analgesia, 75 mg diclofenac sodium was administered via intramuscular route up to three times daily. All patients were discharged postoperative 3.day with oral diclofenac sodium 50 mg three times daily. All patients were hospitalized for two days to assess the need of medications and Visual Analogue Scale. All patients were invited for follow-up assessment postoperative 3. days and then weekly for 4 weeks after the operation.

### 3.4. Pain Evaluation

The Visual Analogue Scale (VAS) was employed as a validated established self-reported measure for pain evaluation (11). Patients were familiarized with an 11-point Visual Analogue Pain Scale scoring from 0 to 10. The patients were asked for pain levels on postoperative 6th, 8th and 24th hours. Amount of consumed analgesics in first 48 hours; pain levels while resting, in first defecation, on 3.day and on first week were recorded.

### 3.5. Wound Healing Evaluation

The patient’s wounds were evaluated at scheduled appointments. Wound healing was defined as degree of epithelialization which is seen on physical examination. The state of the wound was graded according to a five-point scale from sloughy to completely healed ( 12 ) (Table 2). 

**Table 2.** Grading Scale for Wound Healing

Grade	State of wound
**1**	Sloughy
**2**	No granulation
**3**	Granulating
**4**	Epithelializing
**5**	Completely epithelialized

All wound healing evaluations were performed by the same surgeon who did not take part at the operation.

### 3.6. Statistical Analysis

SPSS 18.0 software package was used for statistical analysis. The scores were failed to conform parametric inferential statistics assumptions. A Kolmogorov-Smirnov test showed that none of the dependent variables are normally distributed. Since variance tests depend heavily on these assumptions, an alternative nonparametric Kruskal –Wallis test was used in order to determine whether there are differences between the groups. In accordance with test results, Mann-Whitney U test was used in order to determine source of difference for significant differences. P < 0 .001 value was accepted as significant. Monte Carlo methods were also used for p value due to low sample size.

## 4. Results

Table 1 display that each group has twenty-three patients. Ten (14.5 %) were female and fifty-nine (85.5 %) were male. Patient’s mean age was 41.3 ± 13.0 (19-66) years. There were no statistically significant difference in terms of age or gender (P = 0.345 and P = 0.108, respectively). There were no statistically significant difference between mean number of excised haemorrhoidal piles between patient groups (P = 0.070). All patients had prolapsus. A minimal bleeding was observed in four patients in first the seven-day period. Three patients of them were in Harmonic Scalpel group and one patient was in LigaSure group. No reoperation was required for these patients and their bleeding stopped spontaneously. Gas incontinence on one patient, partial anal stenosis on one patient and recurrence on one patient were observed as late complications in patient group who were operated with LigaSure. No additional pathology was observed for any other patient. 

Mean operative time of patients who were operated with LigaSure or Harmonic Scalpel was significantly shorter when compared with conventional group (P < 0.001). Also, perioperative bleeding rates were significantly higher for conventional surgery (P < 0.001). However, the most considerable differences between the groups were for analgesic requirements in postoperative period, degree of pain according to VAS scale and differences in postoperative wound healing. When degree of pain was compared according to VAS scale, the results of conventional surgery conducted patient group and the Harmonic Scalpel group were close to each other, however degree of pain was significantly higher in LigaSure group (Table 3). (P = 0.741, P = 0.087, P = 0.067 respectively, P < 0.001). 

**Table 3.** Comparison of VAS Score

Grup, n.= 23	Mean ± SD	Min	Max	Percentiles			Z	Monte Carlo Sig. (2-tailed) ^a^
				25th	50th (Median)	75th		
**6 Hours**								
G1	7.52 ± 1.410	4	10	6.0	8.0	8.0	-5.165	0.000 ^b^
G2	4.30 ± 1.460	1	8	4.0	4.0	5.0		
G1	7.52 ± 1.410	4	10	6.0	8.0	8.0	-5.055	0.000 ^b^
G3	4.30 ± 1.550	2	8	3.0	4.0	5.0		
G2	4.30 ± 1.460	1	8	4.0	4.0	5.0	-0.339	0,741
G3	4.30 ±1.550	2	8	3.0	4.0	5.0		
**8 Hours**								
G1	8.30 ± 1.020	6	10	8.0	8.0	8.0	-5.686	0.000 ^b^
G2	4.39 ± 1.699	1	8	3.0	4.0	6.0		
G1	8.30 ± 1.020	6	10	8.0	8.0	8.0	-5.906	0.000 ^b^
G3	3.61 ±1.469	1	7	3.0	3.0	4.0		
G2	4.39 ± 1.699	1	8	3.0	4.0	6.0	-1.703	0,087
G3	3.61 ± 1.469	1	7	3.0	3.0	4.0		
**24 Hours**								
G1	5.13 ± 2.242	3	10	3.0	4.0	7.0	-5.169	0.000 ^b^
G2	2.13 ± 869	1	4	1.0	2.0	3.0		
G1	5.13 ± 2.242	3	10	3.0	4.0	7.0	-5.933	0.000 ^b^
G3	1.39 ± 499	1	2	1.0	1.0	2.0		
G2	2.13 ±.869	1	4	1.0	2.0	3.0	-3.042	0,003 ^c^
G3	1.39 ± .499	1	2	1.0	1.0	2.0		
**3 days**								
G1	3.22 ± 1.976	0	8	2.0	3.0	4.0	-4.003	0.000 ^b^
G2	1.13 ± 1.140	0	4	0.0	1.0	2.0		
G1	3.22 ±1.976	0	8	2.0	3.0	4.0	-5.409	0.000 ^b^
G3	1.39 ± .499	0	1	0.0	0.0	1.0		
G2	1.13 ± 1.140	0	4	0.0	1.0	2.0	-2.409	0,015 ^d^
G3	1.39 ± .499	0	1	0.0	0.0	1.0		
**First Week**								
G1	1.39 ± 1.469	0	4	0.0	1.0	3.0	-3.938	0.000 ^c^
G2	00 ± .000	0	0	0.0	0.0	0.0		
G1	1.39 ± 1.469	0	4	0.0	1.0	3.0	-3.938	0.000 ^c^
G3	00 ± .000	0	0	0.0	0.0	0.0		
G2	00 ± .000	0	0	0.0	0.0	0.0	0.000	
G3	00 ± .000	0	0	0.0	0.0	0.0		
**Resting Pain Levels**								
G1	3.43 ± 1.441	2	7	2.0	3.0	4.0	-3.947	0.000 ^b^
G2	1.87 ± .920	1	4	1.0	2.0	3.0		
G1	3.43 ± 1.441	2	7	2.0	3.0	4.0	-5.413	0.000 ^b^
G3	1.39 ± .499	1	2	1.0	1.0	2.0		
G2	1.87 ± .920	1	4	1.0	2.0	3.0	-1.776	0,067
G3	1.39 ± .499	1	2	1.0	1.0	2.0		
**First Defecation**								
G1	9.65 ± .714	8	10	10.0	10.0	10.0	-4.480	0.000 ^b^
G2	7.09 ± 2.234	3	10	6.0	8.0	9.0		
G1	9.65 ± .714	8	10	10.0	10.0	10.0	-6.026	0.000 ^b^
G3	3.61 ± 1.469	1	7	3.0	3.0	4.0		
G2	7.09 ± 2.234	3	10	6.0	8.0	9.0	-4.498	0,000 ^b^
G3	3.61 ± 1.469	1	7	3.0	3.0	4.0		

^a^Based on 10000 sampled tables

^b^P < 0,001

^c^P < 0,01

^d^P < 0,05

Above, the results of Mann-Whitney U test were presented in order to make a paired comparison between descriptive statistics and groups in accordance with pain values. Monte Carlo method was used for p value due to low sample size (Table 4). 

**Table 4.** Comparison of VAS Score

Time, n = 69	Mean ± SD	Min	Max	Percentiles			z	Monte Carlo Sig. (2-tailed) ^a^
				25th	50th (Median)	75th		
**6 Hours**	5.38 ± 2.108	1	10	4.00	5.00	8.00	-,221 ^b^	0.831
**8 Hours**	5.43 ± 2.500	1	10	3.00	6.00	8.00		
**6 Hours**	5.38 ± 2.108	1	10	4.00	5.00	8.00	-6,357 ^c^	0.000 ^d^
**24Hours**	2.88 ± 2.146	1	10	1.00	2.00	3.50		
**6 Hours**	5.38 ± 2.108	1	10	4.00	5.00	8.00	-6,986 ^c^	0.000
**3.day**	1.58 ± 1.794	0	8	.00	1.00	2.50		
**6 Hours**	5.38 ± 2.108	1	10	4.00	5.00	8.00	-7,246 ^c^	0.000 ^d^
**First Week**	.46 ± 1.065	0	4	.00	0.00	.00		
**8 Hours**	5.43 ± 2.500	1	10	3.00	6.00	8.00	-6,749 ^c^	0.000 ^d^
**24Hours**	2.88 ± 2.146	1	10	1.00	2.00	3.50		
**8 Hours**	5.43 ± 2.500	1	10	3.00	6.00	8.00	-7,091 ^c^	0.000 ^d^
**3.day**	1.58 ± 1.794	0	8	.00	1.00	2.50		
**8 Hours**	5.43 ± 2.500	1	10	3.00	6.00	8.00	-7,243 ^c^	0.000 ^d^
**First Week**	.46 ± 1.065	0	4	.00	0.00	.00		
**24 Hours**	2.88 ± 2.146	1	10	1.00	2.00	3.50	-6,67 ^c^	0.000 ^d^
**3.day**	1.58 ± 1.794	0	8	.00	1.00	2.50		
**24 Hours**	2.88 ± 2.146	1	10	1.00	2.00	3.50	-7,230 ^c^	0.000 ^d^
**First Week**	.46 ± 1.065	0	4	.00	0.00	.00		
**3.day**	1.58 ± 1.794	0	8	.00	1.00	2.50	-5,909 ^c^	0.000 ^d^
**First Week**	.46± 1.065	0	4	.00	0.00	.00		

^a^Based on 10000 sampled tables

^b^Based on negative ranks.

^c^Based on positive ranks

^d^P < 0,001

Wilcoxon test was used instead of paired t test since variables are not normally distributed. The results of Wilcoxon Signed-Rank Test were presented in order to compare descriptive statistics and pain value. Analgesic consumption was least for the patients who were operated with conventional method and was most for patients who were operated with LigaSure (Table 5) (P < 0.001). 

**Table 5.** Comparison of Analgesic Use

Group	n	Mean ± SD	Min	Max	Percentiles			Z	Monte Carlo Sig. (2-tailed) ^a^
					25th	50th (Median)	75th		
**Tramadol Hydrochloride**									
G1	23	782.61 ± 83.406	400	800	800.0	800.0	800.0	-4.047	0.000 ^b^
G2	21	552.38 ± 199.045	400	800	400.0	400.0	800.0		
G1	23	782.61 ± 83.406	400	800	800.0	800.0	800.0	-5.482	0.000 ^b^
G3	19	442.11 ± 126.121	400	800	400.0	400.0	400.0		
G2	21	552.38 ± 199.045	400	800	400.0	400.0	800.0	-1.986	0,070 ^d^
G3	19	442.11 ± 126.121	400	800	400.0	400.0	400.0		
**Diclofenac Sodium**									
G1	23	2.30 ± 1.222	1	5	1.0	2.0	3.0	-3.072	0.003 ^c^
G2	10	1.10 ± .316	1	2	1.0	1.0	1.0		
G1	23	2.30 ± 1.222	1	5	1.0	2.0	3.0	-1.295	0.205 ^d^
G3	11	1.73± .905	1	3	1.0	1.0	3.0		
G2	10	1.10 ± .316	1	2	1.0	1.0	1.0	-1.861	0,110 ^d^
G3	11	1.73 ± .905	1	3	1.0	1.0	3.0		

^a^Based on 10000 sampled tables

^b^P < 0,001

^d^P < 0,05

^c^P < 0,01

The results of Mann-Whitney U test were presented in order to compare the tramadole and diclofenac sodium consumption rates regarding descriptive statistics and groups. Monte Carlo method was used for p value due to low sample size. Wound healing rate was the highest for patients who were operated by conventional method (Table 6) (P < 0.001). 

**Table 6.** Outcomes of Wound Healing

Group, n = 23	Mean ± SD	Min	Max	Percentiles			Z	Monte Carlo Sig. (2-tailed) ^a^
				25th	50th (Median)	75th		
**Wound Completely epithelized by 1th week**								
G1	1.48 ± 511	1	2	1.0	1.0	2.0	-4.845	0.000 ^b^
G2	2.52 ± 511	2	3	2.0	3.0	3.0		
G1	1.48 ± 511	1	2	1.0	1.0	2.0	-5.640	0.000 ^b^
G3	3.35 ± 714	2	4	3.0	3.0	4.0		
G2	2.52 ± 511	2	3	2.0	3.0	3.0	-3.736	0.000 ^b^
G3	3.35 ± 714	2	4	3.0	3.0	4.0		
**Wound Completely Epithelized by 2th week**								
G1	2.65 ± 487	2	3	2.0	3.0	3.0	-3.379	0.001 ^c^
G2	3.13 ± 344	3	4	3.0	3.0	3.0		
G1	2.65± 487	2	3	2.0	3.0	3.0	-5.653	0.000 ^b^
G3	4.00 ± 522	3	5	4.0	4.0	4.0		
G2	3.13 ± 344	3	4	3.0	3.0	3.0	-4.934	0.000 ^b^
G3	4.00± 522	3	5	4.0	4.0	4.0		
**Wound Completely Epithelized by 3th week**								
G1	3.22 ± 422	3	4	3.0	3.0	3.0	-5.343	0.000 ^b^
G2	4.09 ± .288	4	5	4.0	4.0	4.0		
G1	3.22 ± 422	3	4	3.0	3.0	3.0	-5.503	0.000 ^b^
G3	4.48 ± 511	4	5	4.0	4.0	5.0		
G2	4.09 ± 288	4	5	4.0	4.0	4.0	-2.915	0,009 ^c^
G3	4.48 ± 511	4	5	4.0	4.0	5.0		

^a^Based on 10000 sampled tables

^b^P < 0,001

^c^P < 0,01

The results of Mann-Whitney U test were presented in order to make a paired comparison between descriptive statistics and groups for healing values. Monte Carlo method was used for p value due to low sample size (Table 7). 

**Table 7.** Outcomes of Wound Healing

Time, n = 69	Mean ± SD	Min	Max	Percentiles			z	Monte Carlo Sig. (2-tailed) ^a^
				25th	50th (Median)	75th		
**Wound Completely Epithelized**								
1st week	2.45 ± 0.963	1	4	2	2	3	-6,737 ^b^	0.000 ^c^
2nd week	3.26 ± 0.721	2	5	3	3	4		
1st week	2.45 ± 0.963	1	4	2	2	3	-7,228 ^a^	0.000 ^c^
3th week	3.93 ± 0.671	3	5	3	4	4		
2nd week	3.26 ± 0.721	2	5	3	3	4	-6,782 ^a^	0.000 ^c^
3th week	3.93 ± 0.671	3	5	3	4	4		

^a^Based on 10000 sampled tables

^b^Based on negative ranks

^c^P < 0,001

Wilcoxon test was used instead of paired t test since variables are not normally distributed. The results of Wilcoxon Signed-Rank Test were presented in order to compare descriptive statistics and pain value each other for the healing values. Monte Carlo method was used for p value due to low sample size. Repeated measures analysis revealed that, there were statistically significant difference between mean values when sum of three measurements of wound healing was evaluated with respect to groups (Tests of Between-Subjects Effects: F = 83,804; P < 0.001). There were statistically significant difference between results of three measurements of the study without any distinction between groups (Tests of Within-Subjects Effects-factor: Greenhouse-Geisser F = 282,321; P < 0.001). There were statistically significant difference between results three measurements when evaluated with interactions with groups (Tests of Within-Subjects Effects-factor*group: Greenhouse-Geisser F = 7,502; P < 0.001). There were statistically significant difference between mean values when sum of five measurements of pain value was evaluated with respect to groups (Tests of Between-Subjects Effects: F = 85,166; P < 0.001). There were statistically significant difference between results of five measurements of the study without any distinction between group (Tests of Within-Subjects Effects -factor: Greenhouse-Geisser F =288, 348; P < 0.001). There were statistically significant difference between results five measurements when evaluated with interactions with groups (Tests of Within-Subjects Effects-factor*group: Greenhouse-Geisser F = 8,466; P < 0.001).

## 5. Discussion

Haemorrhoidal disease is a common disorder affecting people of all ages and both sexes. It is estimated that 50% of the people older than 50 years old experience haemorrhoidal symptoms at least for a period of time. The causes of haemorrhoidal disease are multiple, but most are attributable to difficulty in defecation or constipation. Over the last years, there has been increasing attention on surgical procedures to treat haemorrhoids. Several comparative studies have been conducted to compare conventional procedures with new surgical techniques, such as haemorrhoidectomy with Harmonic Scalpel or LigaSure to treat grade II, III and IV haemorrhoids (13-15). The ideal method should combine the high safety and efficacy for treatment, yield least postoperative pain and provide comfort to the patient. Conventional haemorrhoidectomy, as first described by Milligan and Morgan, is still the most widely used, effective, and definite surgical treatment for patients with symptomatic grade III and IV haemorrhoids. However, it is associated with significant postoperative complications such as pain, bleeding and mucous discharge. Although a consensus on treatment of grade III and IV haemorrhoids have been established, still there is a debate for optimal technique to minimize postoperative complications (16, 17).

Authors declared different postoperative complication rates after surgical haemorrhoidectomy (18-20). The reported incidence of some postoperative complications varies widely from study to study, primarily as a result of diverse definitions of problems such as postoperative hemorrhage, fecal incontinence, and urinary retention. Postoperative pain is the most distressing concern for the patient after hemorrhoidectomy, and many studies have been conducted regarding various analgesic regimens, operative techniques, and surgical instruments to prevent this important issue. Maurizio G et al. (3) reported anal stenosis in one patient and minor bleeding in one patient among 27 patients they operated by LigaSure method and they observed minor bleeding in three patients among 25 patients operated by conventional method. We observed partial anal stenosis in one patient and minor bleeding in one patient in the group operated by LigaSure method. We did not observe ant significant postoperative bleeding in the patient group operated by conventional method.

Nienhuijs SW et al. (10) and Mastakow MY et al. (21) reported that degree of pain and analgesic consumption rates are less during postoperative period for the patient groups who were operated by LigaSure in their meta-analyses. We observed in our study that degree of pain is less and analgesic consumption rates are lower for the patient group who were operated by conventional method. As Mastakow MY et al. (21) reported in their meta-analysis, we observed that operation time is shorter for Ligasure haemorrhoidectomy. Mastakow MY et al. reported that wound healing rates were better for patient group who were operated by LigaSure method, whereas we observed that wound healing rates were better for patient group who were operated by conventional method. In the study conducted by Waleed O et al. (9) degree of pain and analgesic medication consumption for patient group who were operated by Harmonic Scalpel was less than conventional group during postoperative period. In our study, there were no significant difference between G2 and G3 for postoperative pain according to VAS, but postoperative pain according to VAS in G1 was significantly higher than both other two groups. Analgesic consumption rates of the patients who were operated by conventional method were significantly less than both other two groups. At the same time, wound healing was also significantly better for conventional group.

In conclusion, these new cauterization devices used for haemorrhoid surgery provide some advantages such as reducing operation time and decreasing amount of bleeding. However, as we found, there may be some disadvantages for these devices; degree of pain is higher during postoperative period, analgesic requirement is quite higher and wound healing rates are worse. Moreover, these equipments are for single-use and rather costly. In contrast to mostly claimed, lateral heat dissipation seems to be significant for these devices and as a result of thermal effect, available negative outcomes in our study appear due to tissue damage. Regarding these factors, we suggest that conventional surgical technique for haemorrhoidectomy remains to be safer and more accessible.
